# Analysis of Factors Influencing Food Nutritional Labels Use in Nanjing, China

**DOI:** 10.3390/foods9121796

**Published:** 2020-12-03

**Authors:** Jing Zhang, Liangliang Zhai, Maurice Osewe, Aijun Liu

**Affiliations:** 1 College of Economics & Management, Nanjing Agricultural University, Nanjing 210014, China; 2019106030@njau.edu.cn (J.Z.); 15205172104@163.com (L.Z.); mauriceosewe@gmail.com (M.O.); 2 China Center for Food Security Studies, Nanjing Agricultural University, Nanjing 210014, China

**Keywords:** food nutrition label, Nanjing, food nutrition, binary logistic, diet

## Abstract

A correct use of food nutrition la bels benefits consumers in pursuing balanced diets. As a result, we conducted interviews in Nanjing, China and randomly sampled 427 respondents. Further, we used both descriptive statistics and binary logistic regression to assess their perception of food nutrition label use. The results indicated that the current situation is not ideal in Nanjing. Only 7.26% of respondents use labeling consistently. Data on purchasing experience, comprehension, nutritional value, work sector, chronic disease, diet awareness, nutritional knowledge, and age were statistically significant. Conversely, taste and primary shopper categories negatively influenced food nutrition label use. In light of the above results, we propose policy recommendations to promote consumers’ use of food nutrition labels. These are also beneficial in improving diet and relieving chronic diseases.

## 1. Introduction

People pay less attention to healthy eating habits with improvements in their living standards. This leads to a rising number of individuals suffering from various chronic diseases that have become significant causes of death in China [1]. Particularly, the number of patients who suffer from chronic diseases due to diet amounts to 2.493 million. This accounted for 20% of total chronic disease patients [2]. Thus, there is a need to solve this menace by improving diet, and food nutrition labeling is directly related to dietary habits [2,3]. According to [4], people who use nutrition labels have a healthier diet than those who do not. Besides, nutrition labeling is a vital solution to improving public safety and health. In this article we focus on the packaged food nutritional label.

The packaged food nutrition label is a feature of food packaging with accurate information, including expiration dates, ingredient lists, and nutritional value of foods. It has a positive impact on consumers’ dietary intake, especially that of fat, sodium, and cholesterol [5]. Even more, it influences consumers’ healthy habits through perception and behavior. Using food nutrition labeling frequently decreases purchasing desires for unhealthy food [6]. Foods with labels are considered healthier compared to those not labelled [7]. Consumers limit or increase the intake of certain nutrients while using labels [8,9]. This increases the market sales of health products and augments economic benefits [6].

Consequently, countries have implemented several laws and regulations to promote the widespread use of food nutrition labels. In 1990, the United States enacted the Nutrition Labeling and Education Act, which proposed a dissemination of nutritional food information to consumers. In 2008, China published its first Chinese Food Nutrition Labeling Regulation to strengthen healthy eating habits among consumers [10]. More recently, the Chinese government announced the Healthy China 2020 program to reduce the incidence of chronic diseases by promoting a healthy diet [11]. China has also implemented the national compulsory standard, the General Principles of Nutrition Labeling for Prepackaged Foods, which standardized the nutrition information labeling of various enterprises in 2013. These policies and market promotions have improved the use of food nutrition labels in China. Yet this use is still affected by several subjective and objective factors.

The perception of food nutrition labels influences consumers’ choice and use of particular foodstuffs. The more consumers trust, the more willingness to buy, especially for export products [12]. A better understanding of labels relies on a robust comprehension and calculation ability. People with low ability to process information often perform poorly in food nutrition participation [13]. The majority can perform simple calculations and comparisons between products. Yet their ability to accurately interpret information diminishes with the increase in complexity [14]. Complex label designs also complicate consumers’ understanding [15,16,17]. Further, [8] experimented and concluded that consumers focus on nutrition labels and make healthier purchases when the nutritional format is clear. Nutritional knowledge plays an essential role in the use of nutrition labels. For instance, [18] observed that the level of nutritional knowledge affects the use of nutrition labels significantly. Children rarely use nutrition labels because they have little nutritional knowledge and cannot realize the importance of using food nutrition labels [19,20].

Consequently, different personal characteristics result in various choices and use of nutrition labels. In essence, age, gender, and education are essential factors affecting packaged food labels [17,21,22]. Besides, women use packaged food nutrition labels more than men [23]. Culture also plays a significant role in food package label usage. For example, 3500 consumers from seven different European countries displayed significant differences in their preferences for sugar-free gum [16]. Consumers are more sensitive to product attribution, and thus focus on food types, price, and taste [24]. For instance, [25] noted that South African consumers refuse nutrition labels, owing to taste and price. Additionally, inconsistent label format deters costumers when choosing nutrition labels [26]. Furthermore, [27] noted that the most effective and ineffective ways to urge consumers to choose healthier food is through nutrition scores and reference intakes. [28] added that a warning systems traffic lights format has a more significant impact on children’s use.

Different factors influence nutrition labeling usage in various proportions. However, it is unclear whether these factors will influence and affect Chinese consumers’ choice and use of nutrition labels. As a result, this study investigates the use and influential factors of nutrition labels in Nanjing, China. This article adds to the literature in various ways. First, it discusses the relationship between label formats, personal characteristics, product attribution, cognition level, nutritional knowledge, and nutrition labeling using the logistic model. Second, it gains insights into the determinants of Chinese nutrition labeling usage, and further promotes consumers’ balanced nutritional diet. Lastly, it provides recommendations for the rational use of food nutrition labels in various countries and the effective mitigation of the continuing deteriorating trend, using China as a benchmark.

## 2. Methods and Materials

### 2.1. Data Collection

This dataset was collected in 2014 in two phases. Two Suguo supermarkets along Xiaolingwei Street in Xuanwu District, Nanjing, were selected for a pre-survey. A total of 60 questionnaires were distributed, collected, and modified to improve the content. Between April and July, we selected commercial supermarkets in six major urban districts in Nanjing. We interviewed consumers who use packaged foods in 10 supermarkets concerning “the status and factors influencing the use of food nutrition labels.” The list of supermarkets surveyed is shown in the Table 1. This survey was conducted in Nanjing urban area because, as a famous central city and education base in the developed eastern part of China, it is representative in demography, etc.

A total of 450 questionnaires were sent out to consumers in this survey with a valid sample of 427 responses. The proportion of gender and the age distribution were relatively balanced and the participants differed significantly in marital status, monthly family income, and education.

### 2.2. Questionnaire

The questionnaire contained four parts including personal characteristics, use and perception of food nutrition labels, factors influencing nutrition labels, and suggestions. Individual characteristics comprised gender, age, education, and marital status. Use status was illustrated by frequency of using labels. To understand consumers’ perception of food nutrition labels, we used trust, comprehension, and purchasing experience. The degree of trust and understanding included three levels: not at all, incomplete and complete. The influencing attributes consisted of product attributes, nutrition knowledge, and consumption scenarios. Specifically, product attributes involved price, brand, taste, convenience, nutritional values, and varieties. We tested nutritional knowledge by asking two multiple choice questions. That is, “Can you remember the contents of the balanced diet pyramid for Chinese residents?” and “What is the daily salt intake of healthy adults recommended by the Chinese Nutrition Association?” We measured consumption scenarios using three components. First, participants were asked about their family size, structure, and income. Second, the cost of shopping time was judged by multiple choice question. Third, we used consumers’ dietary awareness, attitude towards the market and high-quality products, and their health status to investigate motivational factors.

### 2.3. Empirical Analysis

We used binary selection models to investigate the use of consumer-packaged food nutrition labels, and to estimate its parameters. Further, we specified the binary logistic regression model mathematically as;

P = F(Y) = 1/(1 + e^−Y^) (1)

where Y is a linear combination of variables X_1_, X_2_, X_3_, … X_n_ that is;

Y = β_0_ + β_1_X_1_ + … + β_n_X_n_ (2)

Therefore, the Logit model used in this paper is specified as;

Logistic (P) = ln(P/1 − P) = β_0_ + β_1_X_1_ + … + β_n_X_n_ + ԑ (3)

where, X_1_, X_2_, … X_n_ represents independent variables, β_0_ is a constant term, but β_1_, β_2_, …, β_n_ are regression coefficients of the respective variables. *P* is the probability that a consumer uses a nutrition label. Considering (1 − P) is the probability that it is not used, then (P/1 − P) is the event occurrence ratio. The dependent variable Y (whether to use food nutrition label) and explanatory variables X (personal characteristics, product attributes, consumption scenarios, nutritional knowledge, and the cognition of the label) are measured in the analysis model. Further, we used the assignment method, i.e., Y is a binary classification variable where consumers using nutrition labels are categorized as 1, and 0 signifies those who do not.

### 2.4. Household Characteristics

This study uses SPSS software to filter variables by partial regression of partial maximum likelihood estimation to test the accuracy and adaptability of the model. Furthermore, we use the back-off method to measure variable screening. Compared with the ratio chi-square test after eliminating the independent variables, the log-likelihood has a more significant effect on the overall coefficient of the regression equation. The final chi-square value of the model is 160.061 with a Sig value less than 0.050. This justifies the use of the logistic regression model. Regarding the Homer-Lemeshow test, the results indicate goodness of fit of the overall regression model. Table 2 reveals personal characteristics of the respondents. The proportions of respondents aged 18–30, 31–40, 41–50, 51–60, and over 61 years old accounted for 26.46%, 28.34%, 23.42%, 15.46%, and 6.32%, respectively. Most of them were also married, highly educated, and with high monthly household income. More than half (58.08%) of the participants had a bachelor’s degree or above. Besides, only 5.62% of the respondents earn less than 4000 yuan per month.

## 3. Results and Discussion

### 3.1. Usage of Food Nutrition Labels

**Figure 1:**
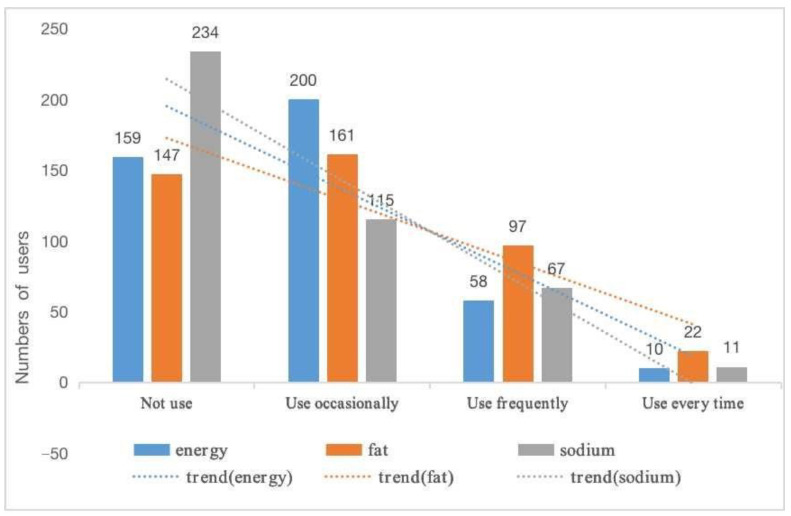
Using Status of three types of nutritional information.

The findings indicate that there is a need to improve the status of food nutrition labels usage. This is because less than 10% (7.26%) of participants use food nutrition labels every time. On the contrary, about a quarter (25.53%) have not yet used them. Besides, 47.31% of the participants use labels sometimes, whereas 19.91% use them frequently. Consumers’ attention to specific nutrients affect their use of food nutrition labels. Figure 1 indicates that most respondents pay insufficient attention to nutritional information. For example, the majority of respondents (54.8%) do not use sodium information completely. Owing to dietary belief and their obesity problem, consumers show more interest in fat information, with a 65.57% use proportion. Concerning frequency of use, the occasional-users preferred energy, obesity, and sodium with proportions 46.84%, 37.7% and 26.93%, respectively. The number of participants who use energy information frequently is reduced by more than four times, accounting only for 13.58%. The other fat and sodium information were used frequently by 22.72% and 15.69% of participants, respectively. Further, 2.34% of participants use energy information whenever shopping. The proportions using fat and sodium information every time account for 5.15% and 2.58%. In other words, the overall situation regarding the use of food nutrition labels for specific information is not optimistic. This restricts the role of the nutrition label in guiding healthy food selection.

**Figure 2:**
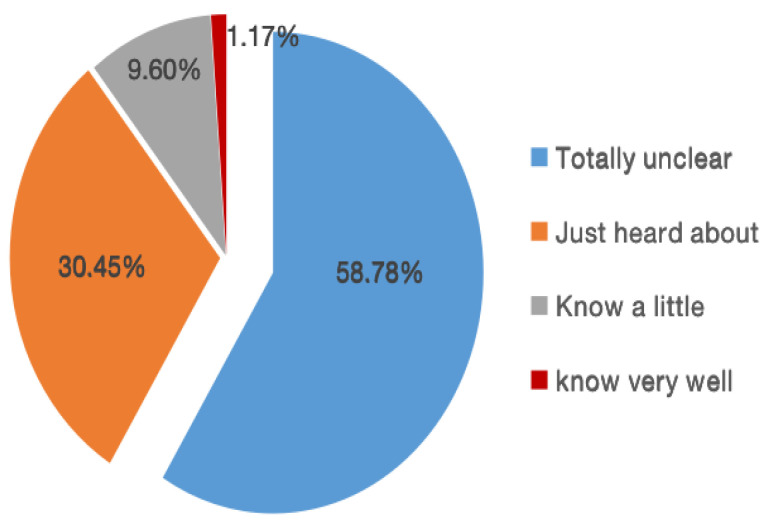
Respondents’ awareness of nutrition label management system.

Consumers in Nanjing are less concerned about the nutrition label management system. Only 1.17% of participants are aware of the General Principles of Nutrition Labeling of Prepackaged Foods (GB 28050-2011). 58.78% of consumers have never heard about this, which could allude to the problems faced by the current management system, such as insufficient information transparency, inadequate publicity, and information asymmetry. It is necessary to strengthen nutrition label education and promotion to encourage consumers to learn more about its management system. This is illustrated in Figure 2.

Generally, consumers in Nanjing lack awareness of food nutrition labels. Almost half were reluctant to believe the information displayed on food packaging and even doubted its authenticity. Therefore, their understanding of data affects their awareness. For instance, 64.87% of the respondents cannot fully understand the information. Regarding past purchasing experience, 60.19% had changed their purchasing decision because of reading nutrition label information.

### 3.2. Regression Results

Using the binary logistic model analysis, the final regression results are presented in Table 3. This shows factors affecting consumers’ use of food nutrition labels.

Taste and major shoppers categories are negatively correlated with the use of food nutrition labels. However, their effects are both low, 0.441, and 0.580, respectively. The use of nutrition labels will adversely drop 0.580 times when major shoppers increase by a unit. If value of taste adds one additional unit, nutrition label usage will fall by 0.441 times. It is seen that purchasing experience and comprehension have a higher significant impact on the use of nutrition labels. An additional unit of similar experience leads to an increase in nutrition label use by 5.529 times. While the unit change in consumers’ comprehension increases food label use by 4.324 times, age has a lower effect on the same. The relevance ratio between age and food nutrition label usage is 1.253. On the other hand, nutritional value, work sector, chronic disease, diet awareness, and nutritional knowledge were all significant factors, with corresponding correlation values of 2.703, 2.634, 2.242, 1.601 and 1.451, respectively.

### 3.3. Discussion

Food nutrition labelling is an essential tool for transferring information on product attributes to the final consumers, though its potential is not entirely explored. The results of this article indicate that food nutrition labels have not been widely used in Nanjing, China. For instance, more than 70% of the participants rarely or never used it. These findings are consistent with the earlier results of Besler et al. [29] that concluded that food nutrition label often confuses customers because of the use of scientific terms. Further, this study show that Chinese consumers’ use of food nutrition labels varies greatly depending on their subjective characters, subjective comprehension, purchasing experience, consumption scenarios, and food attributes. For instance, age plays a vital role in promoting the use of nutrition labels. Elders are more likely to search for nutrition labels because of their nutritional knowledge or health problems. Elders prefer to consider nutritional content, such as sodium and cholesterol. Besides, their purchasing time is much less constrained. Similar outcomes are observed by [30] who noted that elders prefer food nutrition labels because of their health situation and status. The results also show that major household shoppers use fewer labels. This is because low propensity to use food labels depends on how the consumers perceive them [26]. observed that a major segment of consumers believe that nutritional labels are not utterly reliable. Consequently, better comprehension motivates consumers to use nutrition labels continuously. Consumers who understand more label information will use more nutrition labels because they can select specific details quickly and efficiently. This is consistent with most domestic researches. Similar findings are echoed by [8] who documented that understanding the significance of food labels breeds continuous preferences.

Consumers’ purchasing experiences enhance the decision to read nutrition label information. For instance, previous purchasing experience helps consumers perceive the benefits and results of using nutrition labels. This also improves their attention, calculation, and comprehension [24]. Moreover, it encourages consumers to make preferences for brands or stores and this is synonymous with the findings of [25]. Health status and diet awareness have significant positive effects on nutrition label use. Unhealthy consumers pay more attention to food nutrition labels, especially those suffering from chronic diseases. Consumers with stronger diet awareness frequently use food nutrition labels. Therefore, based on the regression results, with a unit increase in the consumer’s diet awareness, nutrition label use will increase by 0.47 times. This is similar to [17] noting that enhancing the efficiency of label use improves the consumers’ nutrition and diet. Different food attributes will lead to various nutrition labels’ use. Taste and food nutritional value have different effect on nutrition label use. In essence, the probability of using labels on good tasting food is lower, due to the overload of existing information [27]. Nutritional food value has a positive effect because consumers usually have a motivation to judge this. As a result, they are willing to spend more time reading the label because they are able to choose a higher food nutritional value through this means. Similarly, while this paper presents significant evidence of the relevant essential policy variables for food nutrition label use, we recognize some limitations. It is not clear how long participants have been using nutrition labels; hence the results are based only on the cross-sectional data. This paper draws results from a small sample of the households, and therefore does not offer a national picture.

## 4. Conclusions

This article sought to explore the factors influencing the use of food nutrition labels in Nanjing, China, using a binary logistic regression model. The results show that only 27.16% of consumers often use nutrition labels. More attention was paid to fat information with 27% of respondents using it frequently when purchasing products. Consumer’s trust and understanding of the information on nutrition labels dwindled because nearly half of the consumers (42%) did not trust the related information, whereas, 64.87% cannot fully understand the label information. Even more, 22.01% reported that they could not completely understand the information. Besides, the majority of consumers (89.23%) were unaware about the relevant laws and management systems. Consequently, cognitive level, product attributes, health perception, nutritional knowledge, and personal characteristics significantly influenced the use of food nutrition labels in Nanjing. In light of the above findings, this article suggests that consumers’ knowledge and understanding can be revised by popularizing basic nutritional knowledge. It is necessary to promote the understanding of nutrition labels directly to enhance consumer trust and the probability of use. The government should also reform relevant laws and systems such as supervising and regulating the nutrition label information of enterprises. Conclusively, this research suggests that more use of nutrition labels can bring much more benefits such as promoting a balanced diet for residents, reducing the prevalence of chronic diseases, and improving public health.

## Figures and Tables

**Table 1:** Regional distribution of questionnaires.

District	Supermarket Surveyed	Number of Samples	Percentage (%)
Xuanwu	Jinrunfa (Ruijin Road)	39	19.44
	Carrefour (Daxinggong)	44	
Qinhuai	Huarun Suguo (Yinlong Garden)	35	17.10
	Wal-Mart (Changle Road)	38	
Jianye	Carrefour (Jiqingmen Street)	46	18.03
	Auchan (Hanzhongmen Street)	31	
Gulou	Lotte Mart (Central Road)	32	17.56
	Jinrunfa (Caochangmen Street)	43	
Xixia	Huarun Suguo (Xianlin Store)	58	13.58
Jiangning	Huarun Suguo (Gold Coast Plaza)	61	14.29

**Table 2:** Personal characteristics of the respondents.

Statistical Indicators	Classification Index	Number of Samples	Proportion (%)
Gender	Male	208	48.71
	Female	219	51.29
Age	18–30	113	26.46
	31–40	121	28.34
	41–50	100	23.42
	51–60	66	15.46
	>61	27	6.32
Education level	Junior high school and below	72	16.86
	high school or technical secondary school	107	25.06
	Undergraduate or college	200	46.84
	Master degree and above	48	11.24
Marital status	Unmarried	99	23.19
	Married	328	76.81
Income(Yuan)	(≤2000)	7	1.64
	(2000, 4000)	17	3.98
	(4000, 6000)	52	12.18
	(6000, 8000)	51	11.94
	(8000, 10,000)	152	35.60
	(≥10,001)	148	34.66

**Table 3:** Regression Analysis Results of Factors Related to Consumer Nutrition Label Reading.

Variables	B	Wald	Sig.	Exp (B)
Comprehension	1.464 ***	23.281	0.000	4.324
Purchasing experience	1.710 ***	19.742	0.000	5.529
Taste	−0.819 ***	6.905	0.009	0.441
Nutritional value	0.994 ***	4.399	0.036	2.703
Major shopper	−0.545 *	3.346	0.067	0.580
Chronic disease	0.807 ***	8.259	0.004	2.242
Diet awareness	0.470 **	5.784	0.016	1.601
Nutritional knowledge	0.372 *	2.932	0.087	1.451
Age	0.225 *	3.705	0.054	1.253
Work sector	0.968 **	5.040	0.025	2.634
Constant	−7.555	36.790	0.000	0.001
Log-likelihood			116.314	
Cox & Snell R Square			0.485	
Nagel kerke R Square			0.679	

Note: *, **, and *** indicate statistical significance at the levels of 10%, 5%, and 1%, respectively.
